# Self-reported physical functioning, cardiometabolic health conditions, and health care utilization patterns in Million Veteran Program enrollees with Traumatic Brain Injury Screening and Evaluation Program data

**DOI:** 10.1186/s40779-022-00435-7

**Published:** 2023-01-03

**Authors:** Alexandra L. Clark, Makenna B. McGill, Erin D. Ozturk, David M. Schnyer, Catherine Chanfreau-Coffinier, Victoria C. Merritt, the VA Million Veteran Program

**Affiliations:** 1 Research Service, VA San Diego Healthcare System (VASDHS), San Diego, CA 92161 USA; 2 Department of Psychology, The University of Texas at Austin, Austin, TX 78712 USA; 3 San Diego State University/University of California San Diego Joint Doctoral Program, San Diego, CA 92120 USA; 4 VA Informatics and Computing Infrastructure (VINCI), VA Salt Lake City Health Care System, Salt Lake City, UT 84148 USA; 5 Department of Psychiatry, School of Medicine, University of California San Diego, La Jolla, CA 29093 USA; 6 Center of Excellence for Stress and Mental Health, VASDHS, San Diego, 92161 USA

**Keywords:** Traumatic brain injury (TBI) screen, CTBIE, Health outcomes, Cardiometabolic health, Veterans, Million Veteran Program (MVP)

## Abstract

**Background:**

Examining the health outcomes of veterans who have completed the United States Veterans Health Administration’s (VHA’s) Traumatic Brain Injury (TBI) Screening and Evaluation Program may aid in the refinement and improvement of clinical care initiatives within the VHA. This study compared self-reported physical functioning, cardiometabolic health conditions, and health care utilization patterns in Million Veteran Program enrollees with TBI Screening and Evaluation Program data (collected between 2007 and 2019), with the goal of enhancing understanding of potentially modifiable health conditions in this population.

**Methods:**

In this observational cohort study, veterans (*n* = 16,452) were grouped based on the diagnostic outcome of the TBI Screening and Evaluation Program: 1) negative TBI screen (Screen^–^); 2) positive TBI screen but no confirmed TBI diagnosis [Screen^+^/ Comprehensive TBI Evaluation (CTBIE)^–^]; or 3) positive TBI screen and confirmed TBI diagnosis (Screen^+^/CTBIE^+^). Chi-square tests and analysis of covariance were used to explore group differences in physical functioning, cardiometabolic health conditions, and health care utilization patterns, and logistic regressions were used to examine predictors of Screen^+/–^ and CTBIE^+/–^ group status.

**Results:**

The results showed that veterans in the Screen^+^/CTBIE^–^ and Screen^+^/CTBIE^+^ groups generally reported poorer levels of physical functioning (*P’s* < 0.001, *n*_*p*_^2^ = 0.02 to 0.03), higher rates of cardiometabolic health conditions (*P’s* < 0.001, *φ* = 0.14 to 0.52), and increased health care utilization (*P’s* < 0.001, *φ* = 0.14 to > 0.5) compared with the Screen^–^ group; however, health outcomes were generally comparable between the Screen^+^/CTBIE^–^ and Screen^+^/CTBIE^+^ groups. Follow-up analyses confirmed that while physical functioning, hypertension, stroke, healthcare utilization, and prescription medication use reliably distinguished between the Screen^–^ and Screen^+^ groups (*P’s* < 0.02, *OR’s* 0.78 to 3.38), only physical functioning distinguished between the Screen^+^/CTBIE^–^ and Screen^+^/CTBIE^+^ groups (*P* < 0.001, *OR* 0.99*)*.

**Conclusions:**

The findings suggest that veterans who screen positive for TBI, regardless of whether they are ultimately diagnosed with TBI, are at greater risk for negative health outcomes, signifying that these veterans represent a vulnerable group that may benefit from increased clinical care and prevention efforts.

**Supplementary Information:**

The online version contains supplementary material available at 10.1186/s40779-022-00435-7.

## Background

It is crucial that we improve our understanding of the physical and psychological consequences of military deployment. United States Veterans involved in the conflicts in Iraq and Afghanistan have returned with unprecedented rates of traumatic brain injury (TBI) and mental health conditions, including posttraumatic stress disorder (PTSD) and depression, which have been linked to increased rates of disability, unemployment, and poorer overall quality of life [1, 2]. Beyond the negative functional impact, these deployment-related conditions are also associated with adverse health conditions and higher health care costs [3, 4]. Research has shown that treatment-seeking veterans with comorbid diagnoses of TBI and PTSD have higher health care utilization and demonstrate a greater number of medical diagnoses (e.g., pain, migraines) and chronic diseases (e.g., hypertension, diabetes) than veterans with TBI or PTSD alone [5–7]. There is also strong evidence to suggest that PTSD negatively impacts health outcomes, although the independent effect of TBI has been difficult to examine given the high degree of psychiatric comorbidity within Veteran samples [8, 9].

While this research has helped inform clinical care initiatives emphasizing targeted medical and behavioral interventions in veterans with comorbid diagnoses, preliminary work from the Veterans Affairs (VA) Million Veteran Program (MVP) suggests a need to look beyond these traditional paradigms of examining outcomes in veterans with TBI, PTSD, and comorbid TBI/PTSD. Importantly, Veterans Health Administration (VHA) directives require that medical providers complete a four-item TBI screening with all Iraq/Afghanistan-era veterans who enroll in the VA and that any veteran with a positive screen subsequently be referred to a TBI specialist for further evaluation [10, 11]. The Comprehensive TBI Evaluation (CTBIE), a semi structured clinical interview, is then conducted by a TBI specialist who assesses TBI injury details (e.g., loss or alteration of consciousness, posttraumatic amnesia) to determine whether a reported injury meets clinical criteria for TBI [11, 12]. This process results in three diagnostic groups: veterans who have 1) a negative TBI screen (Screen^–^); 2) a positive TBI screen, but no subsequent TBI diagnosis on the CTBIE (Screen^+^/CTBIE^–^); or 3) a positive TBI screen and confirmed TBI diagnosis on the CTBIE (Screen^+^/CTBIE^+^).

To date, several studies have leveraged TBI Screening and Evaluation Program data to examine clinical outcomes in this Operation Enduring Freedom/Operation Iraqi Freedom/Operation New Dawn (OEF/OIF/OND) cohort [9, 13–18]. The results from one of these studies conducted within MVP revealed that the Screen^+^/CTBIE^–^ and Screen^+^/CTBIE^+^ groups reported worse cognitive and psychiatric outcomes than the Screen^–^ group, although the Screen^+^ groups were generally comparable to one another on these outcomes [13]. The authors highlight that veterans who initially screen positive for TBI, regardless of whether they are subsequently diagnosed, represent a vulnerable group in need of clinical care [13]. Another study recently investigated neurobehavioral symptom reporting in military veterans who completed the CTBIE and were enrolled in MVP [18]. This study demonstrated that CTBIE^+^ veterans endorsed greater symptoms than CTBIE^–^ veterans. Additionally, they found that veterans whose neurobehavioral symptoms were attributed to comorbid conditions (i.e., behavioral health and TBI) endorsed greater symptoms than other symptom etiology groups (i.e., TBI alone).

Clarifying health outcomes in MVP veterans who underwent the TBI Screening and Evaluation Program may aid in additional refinement and improvement of clinical care initiatives within the VA. Importantly, there is some preliminary evidence to suggest that TBI history may be associated with prolonged cerebrovascular changes that interact with other cardiovascular conditions that promote neurodegenerative cascades [19, 20]. Given the well-established link between vascular risk factors (e.g., hypertension, diabetes, hyperlipidemia) in mid- and late adulthood and poor brain health [21, 22], enhancing our understanding of physical and cardiometabolic health outcomes within the MVP TBI cohort may be especially important in identifying targeted points of intervention or prevention of age-related cognitive decline and impairment.

The purpose of the present study was to characterize health outcomes and utilization patterns in post deployed MVP veterans who underwent the VHA’s TBI Screening and Evaluation Program. We compared subjective ratings of physical functioning, rates of self-reported cardiometabolic health conditions, and patterns of health care utilization across these three TBI screen/CTBIE groups (i.e., Screen^–^; Screen^+^/CTBIE^–^; Screen^+^/CTBIE^+^). Finally, we explored health factors associated with Screen^+/–^ and CTBIE^+/–^ group status. Our goal was to enhance the understanding of modifiable risk factors commonly associated with an increased risk for disability, mortality, and dementia in late life within a nationally representative VA sample.

## Methods

### Participants and procedures

The present study utilized data from MVP, a large-scale national research initiative that investigates how genes, lifestyle factors, psychiatric health, and military-related environmental factors impact Veteran health outcomes. Comprehensive details on the study design and cohort characteristics have previously been described elsewhere [23]. Any veteran in the VHA, the largest integrated health care system within the United States, is eligible for MVP enrollment. To participate, veterans must provide written informed consent, agree to allow MVP investigators access to details of their electronic health record (EHR) data, complete MVP-specific self-report questionnaires, and supply a blood sample for genetic analysis.

MVP was originally approved by the VA’s Central Institutional Review Board (IRB) in 2010 and is actively recruiting and enrolling veteran participants. IRB approval for the present study (conducted under project ‘MVP026’) was obtained in 2019 (Central IRB# 19-03). Only EHR and MVP survey data collected between October 2007 and October 2019 were utilized for the present study. MVP-enrolled veterans were included in this study if they participated in the TBI Screening and Evaluation Program and completed the MVP Baseline Survey (described below). MVP-enrolled veterans were excluded if diagnostic data from the TBI Screening and Evaluation Program were unavailable or incomplete or if pertinent outcome data from the MVP Baseline Survey (i.e., health outcome data) were unavailable or incomplete.

### Data sources

Data sources for all participants in the present study included: 1) EHR data stored within the VA’s Corporate Data Warehouse (CDW) [24], and 2) the MVP Baseline Survey [23]. Sociodemographic information pertaining to age, sex, race/ethnicity, and military service branch was extracted for each participant using both EHR and survey data. TBI Screening and Evaluation Program data were obtained from the EHR, and all other health outcome data were obtained from the MVP Baseline Survey.

#### EHR VA TBI Screening and Evaluation Program data

Beginning in 2007, the VHA implemented the VA TBI Screening and Evaluation Program, which requires that all post deployed Iraq/Afghanistan-era veterans be screened for possible deployment-related TBI [25]. Upon enrollment in the VHA, clinicians (typically a primary care provider) administer the “TBI Clinical Reminder Screen” to every Iraq/Afghanistan-era veteran. This screening consists of 4 sections: 1) identification of injury events(s) (i.e., blast or explosion, vehicular accident/crash, fragment wound or bullet wound above the shoulders, fall); 2) immediate signs/symptoms (e.g., losing consciousness, being dazed, etc.); 3) acute post-concussive symptoms (e.g., memory difficulties, headache, sleep problems, etc.); and 4) current post-concussive symptoms (e.g., memory difficulties, headache, sleep problems, etc.). Any veteran who responds affirmatively to all 4 sections is determined to have a positive TBI screen, which results in a referral to a TBI specialist who then completes the second-level TBI evaluation, referred to as the CTBIE [25]. Any veteran who did not endorse all 4 sections is determined to have a negative TBI screen.

The CTBIE, a semi structured clinical interview, is a more comprehensive assessment that captures historical event details and injury characteristics pertaining to TBI [17]. Clinicians query veterans about mechanisms of injury (i.e., bullet, vehicular, fall, blast); the presence and duration of loss of consciousness (LOC), alteration of consciousness (AOC), and posttraumatic amnesia (PTA); and follow-up care received (e.g., evacuation from theater, medications prescribed, other professional treatment received). Clinicians are then asked to make diagnostic determinations about whether an injury meets clinical criteria for TBI per VA/Department of Defense (DOD) guidelines [26] by answering “yes” (meaning CTBIE^+^) or “no” (meaning CTBIE^–^) to the following question: “Based on the history of the injury and the course of clinical symptoms, did the veteran sustain a TBI during OEF/OIF deployment?”. Providers completing the CTBIE are specifically instructed to render their diagnostic decision based on the presence and duration of estimated LOC, AOC, and PTA.

Extensive research has evaluated the psychometric properties of the TBI screen and CTBIE [25, 27–31]. These studies have generally shown that the TBI screen and CTBIE have moderate-to-good sensitivity but variable specificity.

Study group classification: Using the results of the TBI Screening and Evaluation Program, veterans were classified into the following 3 groups: 1) Screen^–^; 2) Screen^+^/CTBIE^–^; or 3) Screen^+^/CTBIE^+^.

#### MVP baseline survey data

The MVP Baseline Survey was implemented in 2011 and was designed to capture information about demographics (Section A), anthropomorphic and physical features (Section B), military service experience (Section C), physical activity and lifestyle habits (Section D), health status (Section E), medical history (Section F), health care utilization (Section G), and family medical history (Section H). The Baseline Survey is intended to provide additional context to EHR data. Information from Sections B, E, F, and G was used in the present study. The MVP Baseline Survey results have been utilized in other MVP studies exploring sex differences in health-related characteristics, annual trends in body mass index (BMI), and coronavirus disease 2019-related outcomes [32–34]. While some sections of the MVP Baseline Survey consist of empirically validated questionnaires [i.e., Veterans RAND 12 Item Health Survey (VR-12)], this is not the case for other sections (i.e., the medical comorbidity checklist); thus, additional work characterizing the psychometrics of this measure is needed.

### Self-reported psychiatric diagnoses

Veterans were asked to “Check the appropriate box and indicate the year of diagnosis and whether you currently take any medication(s)” for PTSD, Depression, and/or Anxiety/Panic Disorder in Section F.

### Health outcomes

Health-Related Physical Functioning: Veterans completed the VR-12 in Section E, which is a self-report measure of health-related quality of life [35]. The items in the questionnaire reflect various aspects of physical and mental health, including general health perceptions, physical functioning, and role limitations due to physical and emotional problems [36]. The VR-12 can be summarized into 2 domains: a ‘Physical Health Summary Measure’ and a ‘Mental Health Summary Measure’. This study utilized the ‘Physical Health Summary Measure’ to reflect health-related physical functioning (hereafter referred to as ‘VR-12 Physical Functioning’); on this measure, lower scores are indicative of poorer health-related quality of life [35].

Self-Reported Cardiometabolic Health Conditions: Veterans were asked to “Check the appropriate box and indicate the year of diagnosis and whether you are taking any medication(s)” for several cardiometabolic health conditions in Section F. The following 11 health conditions (under the ‘Circulatory Systems Problems’ and ‘Other Conditions’ subsections) were explored in this study: high blood pressure/hypertension, stroke, transient ischemic attack (TIA), heart attack, coronary artery/coronary heart disease (includes angina), peripheral vascular disease, high cholesterol/hyperlipidemia, pulmonary embolism or deep vein thrombosis (DVT), congestive heart failure, other circulatory system problems, and diabetes.

Additionally, height (feet, inches) and weight (pounds) from Section B were used to calculate BMI. The BMI variable was then dichotomized as follows: obese (BMI ≥ 30) vs. not obese (BMI < 30). Finally, we also evaluated a Cardiometabolic Disease Burden variable, defined as the total number of endorsed cardiometabolic health conditions from Section F and obesity (range: 0–12); this variable was dichotomized as follows: 0–2 vs. 3 or more health conditions.

Health care utilization patterns: Veterans answered four questions about health care and medication use in Section G: 1) “In the past year, about how much of your health care did you get at a VA facility (e.g., doctor’s visits, hospitalizations, urgent care visits, or counseling)?”; 2) “In the past year, how many times were you a patient in a hospital overnight or longer?”; 3) “How many prescription medications do you currently receive from a VA and non-VA pharmacy?”; and 4) “How many nonprescription medications do you currently receive from a VA and non-VA pharmacy?”. Responses for question 1 (VA Health Care Use) were dichotomized into ‘0–50%’ and ‘51 –100%’; responses for question 2 (Overnight Hospital Visits) and questions 3 and 4 (Rx medications and Non-Rx medications, respectively) were dichotomized into ‘None’ and ‘1 or more’.

### Statistical analyses

All analyses were conducted with Stata (Stata/MP 15.1, StataCorp LLC, College Station, TX, USA). Chi-square tests were used to explore group differences in categorical sociodemographic characteristics and psychiatric symptom ratings. Analysis of covariance (ANCOVA) controlling for age, sex, race/ethnicity, and PTSD were used to explore group differences in VR-12 physical functioning, and chi-square tests were used to explore group differences in self-reported cardiometabolic health conditions, a cardiometabolic summary variable, and health care utilization variables. Bonferroni multiple comparison corrections (0.05/18 = 0.002) were applied to these omnibus tests. Effect sizes are reported as Cramer’s *V* and phi (*φ*) values for the chi-square tests and as partial eta-squared (*n*_*p*_^*2*^) values for the ANCOVA. Pairwise comparisons effect size interpretations were as follows: phi (*φ*) values: small = 0.10; medium = 0.30; large = 0.50; partial eta-squared (*n*_*p*_^*2*^) values: small = 0.01; medium = 0.06; large = 0.14. Finally, a set of logistic regressions adjusting for sociodemographic variables (age, sex, race/ethnicity, PTSD, and time between TBI screening and MVP Baseline Survey completion) were used to examine predictors of Screen^+/–^ and CTBIE^+/–^ group status.

## Results

### Participant characteristics

Among the enrolled veterans (*n* = 16,452), 65% (*n* = 10,796) screened negative for TBI (Screen^–^); 14% (*n* = 2231) screened positive but did not receive a TBI diagnosis on CTBIE (Screen^+^/CTBIE^–^); and 21% (*n* = 3425) screened positive and received a TBI diagnosis on CTBIE (Screen^+^/CTBIE^+^). Participants were predominantly male (80%, *n* = 13,131) and self-identified as non-Hispanic White (61%, *n* = 10,024). The average time between completing the TBI screen and MVP Baseline Survey within the sample was 1146.90 d [standard deviation (SD) = 1141.41, approximately 3 years], and the median was 1050 d.

Participant sociodemographic characteristics by group are presented in Table 1. The three groups significantly differed by age, sex, race/ethnicity, military branch of service, and psychiatric diagnoses (*P’*s < 0.001). Relative to the Screen^+^ groups (i.e., Screen^+^/CTBIE^–^ and Screen^+^/CTBIE^+^), the Screen^–^ group was older, had a higher proportion of females, was more likely to be White, and largely consisted of veterans who served in the Navy and Air Force. However, veterans in the Screen^–^ group were less likely to endorse a PTSD, depression, or anxiety/panic diagnosis.

**Table 1:** Participant sociodemographic characteristics by diagnostic group [*n*(%)]

Variables	Screen^–^ (*n* = 10,796)*	Screen^+^/CTBIE^–^ (*n* = 2231)*	Screen^+^/CTBIE^+^ (*n* = 3425)*	*P*-value	ES (*V)*
Age at CTBIE				< 0.001	0.08
18–30	2306 (21.4)	434 (21.3)	850 (27.0)		
30–40	2451 (22.7)	518 (25.6)	974 (30.9)		
40–50	3376 (31.3)	665 (32.8)	842 (26.9)		
≥ 50	2646 (24.6)	411 (20.3)	479 (15.2)		
Sex/gender (male)	8113 (75.15)	1951(87.45)	3067 (89.60)	< 0.001	0.16
Race/ethnicity				< 0.001	0.08
White, non-Hispanic	6698 (62.6)	1291 (58.2)	2035 (60.0)		
Black, non-Hispanic	1721 (16.1)	364 (16.4)	372 (10.9)		
Hispanic	766 (7.2)	167 (7.5)	335 (9.9)		
Asian	426 (3.9)	53 (2.4)	104 (3.1)		
Multiracial	610 (5.7)	173 (7.8)	272 (8.0)		
Another race	479 (4.5)	171 (7.7)	273 (8.1)		
Branch of service: Air Force (yes)^#^	2417 (22.4)	222 (9.9)	244 (7.1)	< 0.001	0.18
Branch of service: Army (yes) ^#^	5111 (47.4)	1539 (69.1)	2466 (72.0)	< 0.001	0.22
Branch of service: Marine Corps (yes)^#^	1088 (10.1)	345 (15.5)	605 (17.7)	< 0.001	0.09
Branch of service: Navy (yes)^#^	2766 (25.6)	288 (12.9)	346 (20.7)	< 0.001	0.17
PTSD	2023 (19.1)	1421(64.6)	2574 (75.9)	< 0.001	0.52
Depression	2957 (27.9)	1208 (54.9)	1996 (58.9)	< 0.001	0.29
Anxiety/panic	2131 (20.1)	964 (43.8)	1701 (50.9)	< 0.001	0.29

*Actual *n* for each variable may be less due to missing data. ^#^Not mutually exclusive categories; thus, it is possible for a participant to endorse more than one branch of service. *CTBIE* Comprehensive Traumatic Brain Injury Evaluation, *ES* effect size, *V* Cramer’s *V*, *PTSD* posttraumatic stress disorder

### Health-related physical functioning

An ANCOVA adjusting for age, sex, race/ethnicity, and PTSD diagnosis revealed a significant group difference in physical functioning, *F* (2, 15,594) = 201.37, *P* < 0.001, *n*_*p*_^2^ = 0.03). The Screen^+^/CTBIE^+^ and Screen^+^/CTBIE^–^ groups reported poorer physical functioning than the Screen^–^ group (*P’s* < 0.001, *n*_*p*_^2^ = 0.02 to 0.03), and the Screen^+^/CTBIE^–^ group reported significantly poorer physical functioning than the Screen^+^/CTBIE^+^ group (*P* < 0.001, *n*_*p*_^2^ = 0.002). Adjusted means and standard errors for each group, as well as pairwise comparisons, are reported in Table 2.

**Table 2:** TBI Screen/CTBIE group comparisons of physical functioning ratings, self-reported cardiometabolic health conditions, and health care utilization patterns [*n*(%)]

Variables	Screen^–^ (group 1,* n* = 10,796)	Screen^+^/CTBIE^–^ (group 2, *n* = 2231)	Screen^+^/CTBIE^+^ (group 3, *n* = 3425)	Omnibus test result		Pairwise comparisons			
				*P-*value	ES (*V)*	Summary	*φ*_1-2_	*φ*_1-3_	*φ*_2-3_
Health-related physical functioning									
VR-12 physical functioning (Mean ± Standard error)	44.85 ± 0.11	38.77 ± 0.24	37.95 ± 0.19	< 0.001	0.03^b^	1 > 2 > 3	0.02^a^	0.03^a^	0.002^a^
Cardiometabolic health conditions									
High blood pressure/hypertension*	3153 (29.2)	756 (33.9)	1065 (31.1)	< 0.001	0.04	2 > 3 > 1	0.17	0.04	0.06
Stroke	62 (0.6)	39 (1.6)	58 (1.7)	< 0.001	0.06	2 & 3 > 1	0.29	0.33	< 0.001
TIA	70 (0.7)	25 (1.1)	41 (1.2)	0.002	0.03	—	0.05	0.08	< 0.001
Heart attack	137 (1.3)	45 (2.0)	54 (1.6)	0.020	0.02	—	—	—	—
Coronary artery/heart disease	237 (2.2)	65 (2.9)	77 (2.3)	0.120	0.02	—	—	—	—
Peripheral vascular disease	55 (0.5)	20 (0.9)	24 (0.7)	0.070	0.02	—	—	—	—
High cholesterol/hyperlipidemia*	3462 (32.1)	831 (37.3)	1122 (32.8)	< 0.001	0.04	2 > 1 & 3	0.19	0.004	0.16
Pulmonary embolism/DVT	155 (1.4)	45 (2.0)	59 (1.7)	0.100	0.01	—	—	—	—
Congestive heart failure	67 (0.6)	29 (1.3)	27 (0.8)	0.003	0.03	—	—	—	—
Other circulatory problems	275 (2.6)	123 (5.5)	133 (3.9)	< 0.001	0.06	2 > 3 > 1	0.48	0.14	0.11
Diabetes*	843 (7.8)	200 (8.9)	242 (7.1)	0.030	0.02	—	—	—	—
Obesity (BMI > 30)*									
Not obese	6773 (64.1)	1277 (58.8)	1887 (56.6)						
Obese	3798 (35.9)	894 (41.2)	1449 (43.4)	< 0.001	0.07	2 & 3 > 1	0.19	0.52	0.04
Cardiometabolic summary variable*									
Cardiometabolic disease burden									
0–2 conditions	9056 (85.7)	1743 (80.3)	2812 (84.3)						
3 or more conditions	1515 (14.3)	428 (19.7)	524 (15.7)	< 0.001	0.05	2 > 1 & 3	0.36	0.03	0.20
VA health care utilization*									
VA health care use									
0–50%	4444 (41.6)	562 (25.5)	883 (26.1)						
51–100%	6242 (58.4)	1640 (74.5)	2501 (73.9)	< 0.001	0.15	2 & 3 > 1	> 0.50	> 0.50	0.003
Overnight hospital visits									
None	9242 (90.9)	1689 (81.9)	2538 (80.2)						
1 or more	931 (9.1)	373 (18.1)	626 (19.8)	< 0.001	0.14	2 & 3 > 1	> 0.50	> 0.50	0.03
Rx medications									
None	3154 (30.4)	282 (13.0)	460 (13.8)						
1 or more	7213 (69.6)	1887 (87.0)	2882 (86.2)	< 0.001	0.19	2 & 3 > 1	> 0.50	> 0.50	0.009
Non-Rx medications									
None	8637 (88.0)	1552 (78.4)	2446 (79.8)						
1 or more	1181 (12.0)	428 (21.6)	621 (20.3)	< 0.001	0.12	2 & 3 > 1	> 0.50	0.14	0.003

*Actual *n* for each outcome of interest may be less due to missing data. ^§^ VR-12 = lower scores are indicative of worse health-related quality of life. Adjusted group means (age, sex, race/ethnicity, and PTSD) for the VR-12 data are reported in the table

^a^Values are *η*_*p*_^*2*^. ^b^ Values is ES (*η*_*p*_^*2*^). *P*-values < 0.002 represent significant omnibus tests that survived Bonferroni multiple comparisons corrections. Pairwise comparisons effect size interpretations: Partial eta-squared (*η*_*p*_^*2*^) values, small = 0.01, medium = 0.06, large = 0.14; Phi (*φ*) values, small = 0.10, medium = 0.30, large = 0.50. *CTBIE* Comprehensive Traumatic Brain Injury Evaluation, *TBI* traumatic brain injury, *ES* effect size, *V* Cramer’s *V*, *φ* Phi effect size, *η*_*p*_^*2*^ partial eta-squared effect size, *VR-12* Veterans RAND 12 Item Health Survey, *TIA* transient ischemic attack, *DVT* deep vein thrombosis, *BMI* body mass index, *MetS* metabolic syndrome, *VA* Veterans Affairs, *Rx* prescription

### Cardiometabolic health conditions

Chi-square analyses revealed significant group differences in 5 of the 12 cardiometabolic health conditions: hypertension, stroke, hyperlipidemia, other circulatory problems, and obesity (*P’s* < 0.001, *V* = 0.03 to 0.07). The results of the omnibus group and pairwise comparisons are reported in Table 2. Relative to the Screen^–^ group, the Screen^+^/CTBIE^–^ group demonstrated significantly higher rates of these five conditions (*P’s* < 0.001, *φ* = 0.17 to 0.48). Additionally, relative to the Screen^–^ group, the Screen^+^/CTBIE^+^ group demonstrated significantly higher rates of stroke, other circulatory problems, and obesity (*P’s* < 0.001, *φ* = 0.14 to 0.52). There was also a significant group difference in hypertension (*P* = 0.035), but examination of effect sizes revealed that these rates were relatively comparable (*φ* = 0.04).

When comparing the two Screen^+^ groups, the Screen^+^/CTBIE^–^ group demonstrated significantly higher rates of hyperlipidemia (*P* = 0.001) and other circulatory problems (*P* = 0.004) than the Screen^+^/CTBIE^+^ group, with small effect sizes (*φ* = 0.11 to 0.16). There was also a significant group difference in rates of hypertension (*P* = 0.028), but the effect size (*φ* = 0.06) revealed relatively comparable rates between the groups.

Finally, chi-square analyses revealed significant group differences for the cardiometabolic disease burden (*P’s* < 0.001, *V* = 0.05). Relative to the Screen^+^/CTBIE^–^ group, the Screen^−^ and Screen^+^/CTBIE^+^ groups demonstrated significantly lower rates of three or more cardiometabolic conditions, with effect sizes in the small to medium range (*φ* = 0.20 to 0.36).

### VA health care utilization

Chi-square analyses revealed significant group differences in rates of VA health care use, overnight hospital visits, and medication use (*P’s* < 0.001, *V* = 0.12 to 0.19). The Screen^+^/CTBIE^–^ and Screen^+^/CTBIE^+^ groups reported higher rates of VA health care use, hospital visits, and prescription and nonprescription medication use than the Screen^–^ group (*P’s* < 0.001, *φ* = 0.14 to > 0.5). However, these health care utilization rates did not significantly differ between the two Screen^+^ groups (*P’s* > 0.05, *φ* = 0.002 to 0.030). The results of omnibus group and pairwise comparisons are reported in Table 2.

### Health-related predictors of TBI screening and CTBIE group status

#### TBI screening group status

**Fig. 1:**
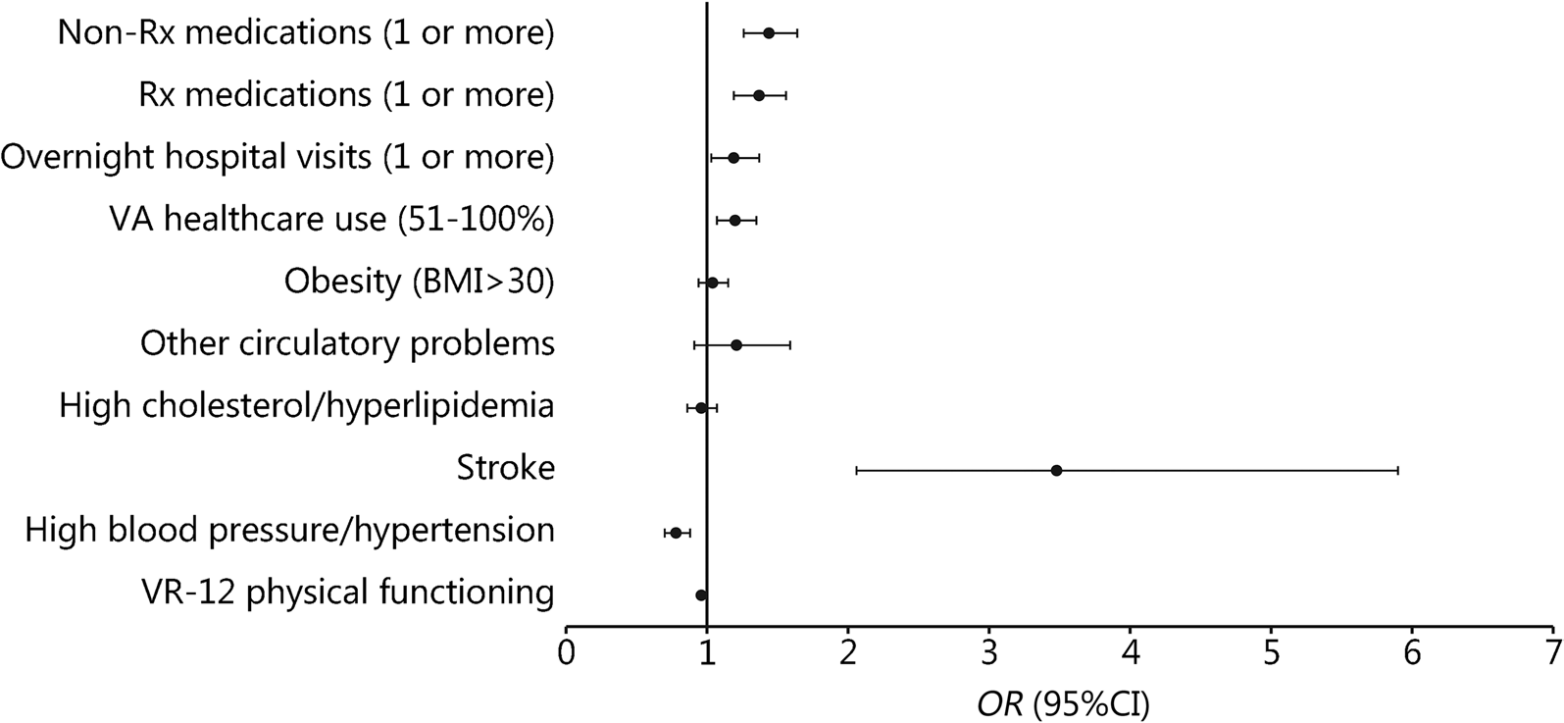
Forest plot of odds ratios (*OR*) for health variables that distinguish between the Screen^–^ vs. Screen^+^ groups in logistic regression analyses. Rx prescription, VA Veterans Affairs, BMI body mass index, VR-12 Veterans RAND 12 Item Health Survey

Given that the groups screening positive for TBI (i.e., Screen^+^/CTBIE^–^ and Screen^+^/CTBIE^+^) generally demonstrated a similar pattern of differences relative to the Screen^–^ group, the two Screen^+^ groups were combined for this comparison. Logistic regression was performed to ascertain which of the significant health outcome variables from the above analyses significantly discriminated between the Screen^–^ and Screen^+^ groups when accounting for age, sex, race/ethnicity, PTSD, and time between TBI screen and MVP Baseline Survey completion. The adjusted logistic regression model was statistically significant, *χ*^2^ (15) = 4003.75, *P* < 0.001, and explained 27.5% of the variance in Screen^+^/^–^ group status. See Fig. 1 for a detailed examination of how the Screen^–^ and Screen^+^ groups differed across health outcomes. Compared with the Screen^–^ group, the Screen^+^ group was significantly more likely to endorse a history of stroke [*P* < 0.001, *odds ratio* (*OR*) = 3.48] and experience higher rates of VA health care use (*P* = 0.001*, OR* = 1.20), hospital visits (*P* = 0.022*, OR* = 1.19), and prescription and nonprescription medication use (*P’s* < 0.001*, OR’s* = 1.37 to 1.44). Additionally, the Screen^+^ group was significantly less likely to report better physical functioning (*P* < 0.001, *OR* = 0.96) and endorse hypertension (*P* < 0.001, *OR* = 0.78). The results from the logistic regression (*OR*, 95% CI, and *P*-values) are reported in Table 3 (see Additional file 1: Table S1 for full results).

**Table 3:** Logistic regression results for associations between health outcomes and TBI Screening and Evaluation Program diagnostic groups

Variables of interest	Model 1: Screen^–^ vs. Screen^+^		Model 2: CTBIE^–^ vs. CTBIE^+^	
	*OR* (95% CI)	*P*-value	*OR* (95% CI)	*P*-value
VR-12 physical functioning	0.96 (0.96–0.97)	< 0.001	0.99 (0.98–0.99)	< 0.001
High blood pressure/hypertension	0.78 (0.70–0.88)	< 0.001	0.97 (0.82–0.99)	0.732
Stroke	3.48 (2.06–5.90)	< 0.001	1.05 (0.60–1.83)	0.860
High cholesterol/hyperlipidemia	0.96 (0.86–1.07)	0.477	0.89 (0.76–1.06)	0.208
Other circulatory problems	1.21 (0.91–1.59)	0.188	0.82 (0.57–1.18)	0.299
Obesity (BMI > 30)	1.04 (0.94–1.15)	0.442	1.10 (0.96–1.28)	0.172
VA health care use (51–100%)	1.20 (1.07–1.35)	0.001	0.91 (0.76–1.08)	0.303
Overnight hospital visits (1 or more)	1.19 (1.03–1.37)	0.022	1.06 (0.88–1.28)	0.546
Rx medications (1 or more)	1.37 (1.19–1.56)	< 0.001	0.83 (0.66–1.03)	0.093
Non-Rx medications (1 or more)	1.44 (1.26–1.64)	< 0.001	0.91 (0.76–1.08)	0.271

All models are adjusted for age group (18–29; 30–39; 40–49; and ≥ 50), sex (male; female); race/ethnicity (White, Non-Hispanic; Black, Non-Hispanic; Hispanic; Asian; Multiracial; Another Race/Ethnicity); and PTSD diagnosis (yes; no). Logistic regression was used to estimate the odds of being classified into the Screen^+^ group for Model 1 (*n* = 13,008) and the CTBIE^+^ group for Model 2 (*n* = 4135) as a function of health outcomes. The Screen^–^ and CTBIE^–^ groups served as the reference group in Models 1 and 2, respectively. *CTBIE* Comprehensive Traumatic Brain Injury Evaluation, *VR-12* Veterans RAND 12 Item Health Survey, *BMI* body mass index, *VA* Veterans Affairs, *Rx* prescription, *OR* odds ratio, *CI* confidence interval

#### CTBIE group status

**Fig. 2:**
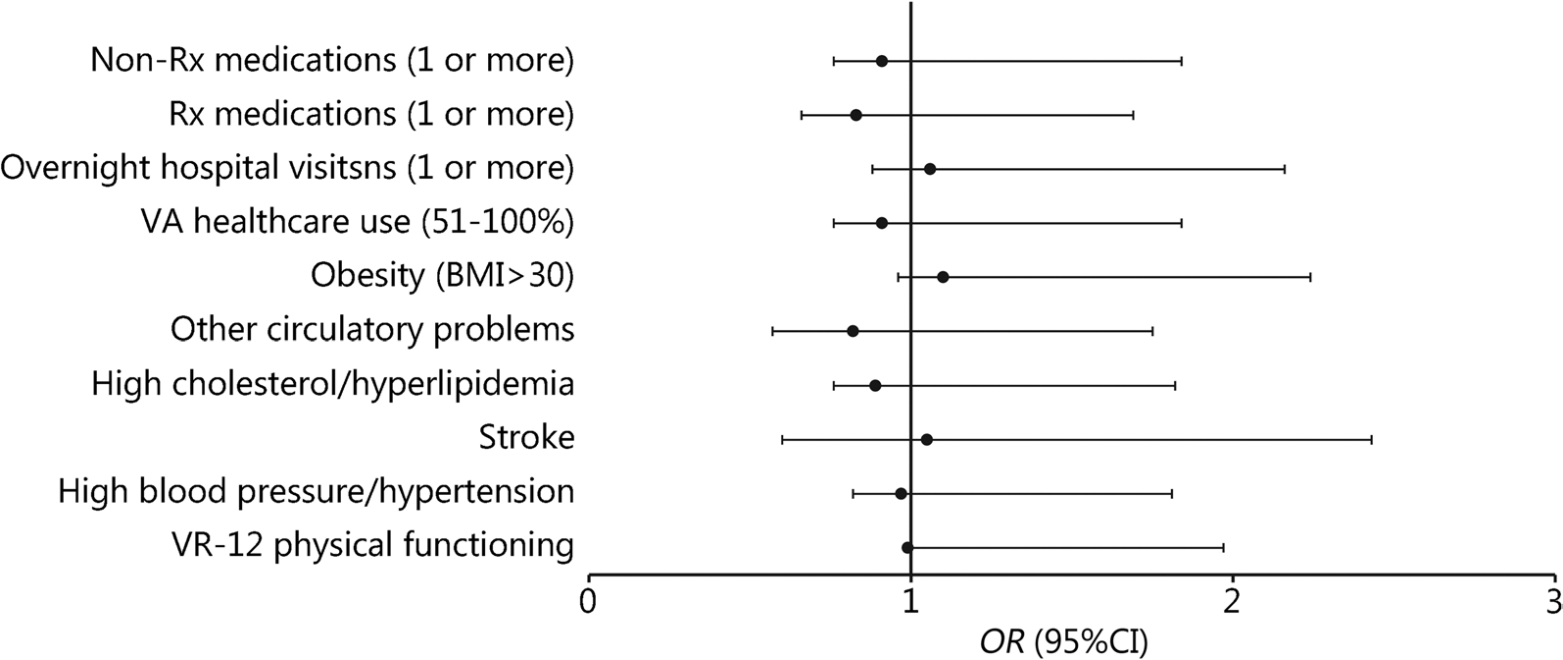
Forest plot of odds ratios (*OR*) for health variables that distinguish between the Screen^+^/CTBIE^–^ vs. Screen^+^/CTBIE^+^ groups in logistic regression analyses. CTBIE Comprehensive Traumatic Brain Injury Evaluation, Rx prescription, VA Veterans Affairs, BMI body mass index, VR-12 Veterans RAND 12 Item Health Survey

To ascertain which of the significant health outcome variables from the above analyses significantly discriminated between the Screen^+^/CTBIE^–^ and Screen^+^/CTBIE^+^ groups, another logistic regression analysis was conducted accounting for age, sex, race/ethnicity, PTSD, and time between TBI screen and MVP Baseline Survey completion. The adjusted logistic regression model was statistically significant, *χ*^2^ (15) = 110.80, *P* < 0.001, but explained only 2.4% of the variance in CTBIE diagnostic group status. See Fig. 2 for a detailed examination of how the Screen^–^/CTBIE^−^ and Screen^+^/CTBIE^+^ groups differed across health outcomes. Relative to the Screen^+^/CTBIE^–^ reference group, the Screen^+^/CTBIE^+^ group was significantly less likely to report better physical functioning, although the effect size of this association is inconsequential (*P* < 0.001*, OR* = 0.99). The results from the logistic regression (*OR*, 95% CI, and *P*-values) are reported in Table 3 (see Additional file 1: Table 2 for full results).

## Discussion

The purpose of the present study was to characterize health outcomes and utilization patterns in MVP veterans who underwent the VHA’s TBI Screening and Evaluation Program. The results revealed that veterans in the Screen^+^/CTBIE^–^ and Screen^+^/CTBIE^+^ groups generally reported poorer levels of physical functioning, higher rates of cardiometabolic health conditions, and increased health care utilization relative to the Screen^–^ group; in contrast, these health outcomes were relatively comparable between the Screen^+^/CTBIE^–^ and Screen^+^/CTBIE^+^ groups. Follow-up logistic regression analyses controlling for sociodemographic factors explored specific health outcomes associated with TBI screen status (i.e., Screen^–^ vs. Screen^+^) as well as CTBIE status (i.e., CTBIE^–^ vs. CTBIE^+^). These results revealed that stroke history and medication use were most associated with an increased likelihood of Screen^+^ group status. However, when examining health outcomes that could discriminate between CTBIE^–^ and CTBIE^+^ group status, we found that most health outcome variables were not significantly associated with group status. The results illustrate that veterans enrolled in MVP who screen positive for TBI, regardless of whether they are subsequently diagnosed with a TBI during a comprehensive clinical evaluation, are at increased risk for negative health outcomes. Taken together, these findings suggest that intervention and health-policy efforts requiring a positive TBI history for the qualification of clinical services may lead to the exclusion of a vulnerable group (i.e., Screen^+^/CTBIE^–^) in need of medical care and continued monitoring.

Our findings are consistent with several other studies demonstrating higher rates of medical disease burden and health care utilization among treatment-seeking Iraq/Afghanistan veterans with a history of TBI and PTSD [5, 6, 37]. This research has shown that veterans with comorbid diagnoses (i.e., TBI/PTSD) or symptom comorbidity clusters (i.e., the Polytrauma Clinical Triad) typically demonstrate the worst health outcomes and that mental health diagnoses are linked to increased risk for cardiovascular risk factors [6, 38]. Given that a large proportion of our Screen^+^/CTBIE^–^ and Screen^+^/CTBIE^+^ groups endorsed psychiatric disorders (44–76%), it is possible that increased rates of cardiovascular health outcomes and health care utilization observed in the Screen^+^ groups are a consequence of these mental health conditions. However, our follow-up logistic regression analyses showed that when controlling for PTSD (Additional file 1: Table S2), several health outcomes of interest were still predictive of Screen^+^ group status. In other words, while PTSD may play an important role, it does not fully account for the observed pattern of results within the Screen^+^ groups.

While several studies have used advanced statistical methods to identify unique clinical phenotypes [e.g., Polytrauma Clinical Triad (TBI, PTSD, and pain) or Deployment Trauma Phenotype (TBI, PTSD, and depression)] in veterans [39, 40], the emergence of these distinct clusters is subject to regional variations in sample characteristics and necessitates that health care providers adequately assess and code for all of these conditions in electronic health records. An advantage of using the VHA TBI screen as an anchor for assessing risk for poor health outcomes is that nearly every veteran involved in OEF/OIF/OND who seeks care at a VA facility should have a completed screening, and a positive screen could simply guide clinicians to engage in continued medical, psychological, and behavioral monitoring of veterans. While we recognize that there are a considerable number of costs and challenges associated with screening for remote TBI [41], our results highlight that these already-collected TBI screening and evaluation data can be easily used for proactive monitoring of veterans’ health over time.

While both the Screen^+^/CTBIE^–^ and Screen^+^/CTBIE^+^ groups generally had worse health outcomes than the Screen^–^ group, differences in the general pattern of health outcomes observed between the two Screen^+^ groups were minimal. Notably, our follow-up logistic regressions that adjusted for important sociodemographic variables showed that only approximately 2% of the variance in CTBIE group status was explained by these health outcome variables, and examination of *ORs* for the statistically significant variables (e.g., *OR* = 0.99 for physical functioning) suggests that these effects are likely not clinically meaningful. In contrast, approximately 28% of the variance in TBI screening group status was explained by these health outcomes, suggesting that the TBI screening is beneficial for detecting veterans who may be at risk for poor long-term physical functioning, cardiometabolic conditions, and increased health care utilization. While we recommend comprehensive assessment and treatment of all cardiometabolic health outcomes, clinicians may want to generally focus on the prevention of stroke within Screen^+^ Veterans.

Somewhat unexpectedly, hyperlipidemia and obesity did not discriminate between the Screen^–^ and Screen^+^ groups, and hypertension was in the opposite direction (i.e., veterans with hypertension were less likely to fall in the Screen^+^ group). This could be associated with the fact that the Screen^–^ group was slightly older and, therefore, likely to be at increased risk for these specific vascular risk factors, given that vascular senescence is thought to start occurring in mid-life [42]. Furthermore, it is important to note that while overall prevalence rates of cardiometabolic conditions were relatively low in this OEF/OIF/OND sample of veterans, as Screen^+^ Veterans continue to age, we suspect that they may be at increased risk for these conditions and continued prevention management may ultimately lead to better late-life functional outcomes. Finally, it is important to highlight that our findings also align with another recent MVP study utilizing this three-group paradigm that similarly found that the two Screen^+^ groups (i.e., Screen^+^/CTBIE^–^ and Screen^+^/CTBIE^+^) endorsed higher rates and worse levels of subjective cognitive impairment than the Screen^–^ group, but cognitive outcomes between the two Screen^+^ groups were minimal [13]. Taken together, the results suggest that a positive TBI screen, regardless of whether a veteran ultimately receives a TBI diagnosis, warrants additional monitoring and clinical care.

A final noteworthy finding that deserves additional consideration is that our results suggest that the TBI screen appears to do a better job at predicting poor health outcomes than the CTBIE. Although our study did not address why this might be the case, it is reasonable to speculate that patient illness perception, repeated assessment of TBI, and/or potential residual side effects associated with subconcussive events may be relevant factors worth considering. For example, research has shown that in a large group (*n* > 1000) of treatment-seeking primary care patients, higher levels of negative illness perceptions (e.g., “I think my health problems could affect the way others see me”; “My health problems make me feel afraid”) were associated with poorer long-term physical health outcomes and this association was strongest among patients with medically unexplained symptoms at 3-, 6-, and 24-month follow-up visits [43]. Additionally, other researchers have raised concerns that repeated TBI screening or assessment many months after an initial injury event may have unintended iatrogenic consequences that could lead to the false attribution of these nonspecific symptoms [44, 45]; however, an alternative possibility is that retrospective recall bias may have led to the underestimation of injury details in the Screen^+^/CTBIE^–^ group that may have contributed to a potential undercoding of genuine TBI events. Finally, there is some evidence to suggest that subconcussive impacts or blast-related events may be linked to neural changes that may explain residual symptoms [46, 47], which could similarly lead to long-term health complications. Additional studies are needed to further tease apart potential mechanisms underlying the negative health outcomes observed in the Screen^+^ groups and to clarify the role of remote injury detail estimation in the coding of TBI events.

There are several limitations to our study that warrant careful consideration. It is important to note that the CTBIE and MVP surveys were completed by veterans who were likely in the chronic phase of injury. Thus, verifying these self-reported injury details as well as determining the exact amount of time between the TBI event and date of CTBIE completion is difficult. Similarly, since the present study was based on retrospective, cross-sectional, medical record data, it is also subject to potential inaccuracies related to the charting and documentation of TBI. While we controlled for time between TBI screening and MVP Baseline Survey completion in our regression analyses, it is important to note that there may be person-to-person variability in the time between assessments. Future longitudinal studies are needed to more carefully characterize the time between TBI and the onset of medical comorbidities in this population. Nevertheless, our study demonstrates important findings regarding health outcomes in a large, nationwide sample of veterans enrolled in the VA’s MVP. Finally, we highlight that this sample is more racially/ethnically diverse, with 40% of the sample being nonwhite.

## Conclusions

Our study revealed that self-reported levels of physical functioning, rates of cardiometabolic health conditions, and VA health care utilization patterns differ as a function of MVP VHA TBI screening and CTBIE group status. Understanding the mechanisms that underlie these differences, as well as how coding of TBI may influence findings, will be important next steps in this line of research. Ultimately, the VHA TBI screening efforts were implemented to identify, treat, and further develop clinical initiatives that better serve veterans. The results from this study highlight that these large-scale efforts can easily be translated into targeted health assessments for further prevention of adverse long-term outcomes.

## Supplementary Information

**Additional file 1: Table S1**. Full logistic regression results for Model 1: associations between health outcomes and TBI Screen^–^ and Screen^+^ groups adjusting for sociodemographic characteristics (*n* = 13,008). **Table S2** Full logistic regression results for Model 2: associations between health outcomes and Screen^+^/CTBIE^–^ and Screen^+^/CTBIE^+^ groups adjusting for sociodemographic characteristics (*n* = 4135).

## Data Availability

The datasets generated and/or analyzed during the current study are not publicly available due to VA restrictions, but the corresponding author is willing to engage with reasonable requests and answer questions about the present study.

## Abbreviations

ANCOVA: Analysis of covariance
AOC: Alteration of consciousness
BMI: Body mass index
CDW: Corporate Data Warehouse
CI: Confidence interval
CTBIE: Comprehensive TBI Evaluation
DOD: Department of Defense
DVT: Deep vein thrombosis
EHR: Electronic health record
ES: Effect size
IRB: Institutional Review Board
LOC: Loss of consciousness
MetS: Metabolic syndrome
MVP: Million Veteran Program
OEF/OIF/OND: Operation Enduring Freedom/Operation Iraqi Freedom/Operation New Dawn
OR: Odds ratio
PTA: Posttraumatic amnesia
PTSD: Posttraumatic stress disorder
Rx: Prescription
SD: Standard deviation
SE: Standard error
TBI: Traumatic brain injury
TIA: Transient ischemic attack
*V*: Cramer’s *V*
VA: Veterans Affairs
VHA: Veterans Health Administration
VR-12: Veterans RAND 12 Item Health Survey
