# Supplementary material for: Self-reported physical functioning, cardiometabolic health conditions, and health care utilization patterns in Million Veteran Program enrollees with Traumatic Brain Injury Screening and Evaluation Program data
Source: Mil Med Res. 2023 Jan 3;10:2. doi: 10.1186/s40779-022-00435-7 (PMC9810242; doi:10.1186/s40779-022-00435-7)
Supplement: Supplementary file 1 — Additional file 1: Table S1. Full logistic regression results for Model 1: associations between health outcomes and TBI Screen– and Screen+ groups adjusting for sociodemographic characteristics (n = 13,008). Table S2 Full logistic regression results for Model 2: associations between health outcomes and Screen+/CTBIE– and Screen+/CTBIE+ groups adjusting for sociodemographic characteristics (n = 4135). [file 40779_2022_435_MOESM1_ESM.pdf]

Table S1 Full logistic regression results for Model 1: associations between health outcomes and TBI Screen^{-} and Screen^{+} groups adjusting for sociodemographic characteristics (n = 13,008)

Variables of interest	*OR* (95% CI)	Standard error	*Z*	*P*-value
Age	0.77 (0.79 – 0.87)	0.02	-9.76	< 0.001
Sex/gender	3.67 (3.29 – 4.26)	0.26	18.52	< 0.001
Race/ethnicity	1.06 (1.03 – 1.10)	0.02	3.81	< 0.001
PTSD diagnosis	9.20 (8.22 – 9.91)	0.47	43.47	< 0.001
Time between TBI screening and baseline survey	0.99 (0.99 – 0.99)	0.000	-10.16	< 0.001
VR-12 physical functioning	0.96 (0.96 – 0.97)	0.002	-16.31	< 0.001
High blood pressure/hypertension	0.78 (0.70 – 0.88)	0.05	-4.07	< 0.001
Stroke	3.48 (2.05 – 5.90)	0.94	4.64	< 0.001
High cholesterol/hyperlipidemia	0.96 (0.86 – 1.07)	0.06	-0.71	0.477
Other circulatory problems	1.21 (0.91 – 1.59)	0.17	1.32	0.188
Obesity (BMI > 30)	1.04 (0.94 – 1.15)	0.05	0.77	0.442
VA health care use (51 – 100%)	1.20 (1.07 – 1.35)	0.07	3.18	0.001
Overnight hospital visits (1 or more)	1.19 (1.03 – 1.37)	0.09	3.26	0.022
Rx medications (1 or more)	1.37 (1.19 – 1.56)	0.09	4.63	< 0.001
Non-Rx medications (1 or more)	1.44 (1.25 – 1.64)	0.06	5.15	< 0.001

Logistic regression was used to estimate the odds of being classified into the Screen^{+} group as a function of health outcomes, adjusting for covariates (age, sex/gender, race/ethnicity, and PTSD diagnosis). The Screen^{-} group served as the reference group. *OR* odds ratio, *TBI* traumatic brain injury, *PTSD* posttraumatic stress disorder, *VR-12* Veterans RAND 12 Item Health Survey, *BMI* body mass index, *VA* Veterans Affairs, *Rx* prescription, *CI* confidence interval

Table S2 Full logistic regression results for Model 2: associations between health outcomes and Screen^{+}/CTBIE^{-} and Screen^{+}/CTBIE^{+} groups adjusting for sociodemographic characteristics (n = 4135)

Variables of interest	*OR* (95% CI)	Standard error	*Z*	*P*-value
Age	0.83 (0.78 – 0.91)	0.03	-4.46	< 0.001
Sex/gender	1.27 (1.02 – 1.59)	0.14	2.15	0.031
Race/ethnicity	0.98 (0.94 – 1.02)	0.02	-1.01	0.313
PTSD diagnosis	1.77 (1.53 – 2.07)	0.14	7.10	< 0.001
Time between TBI screening and baseline survey	0.99 (0.99 – 0.99)	0.00	-3.13	0.002
VR-12 physical functioning	0.99 (0.98 – 0.99)	0.003	-3.96	< 0.001
High blood pressure/hypertension	0.97 (0.82 – 0.99)	0.08	-0.34	0.732
Stroke	1.05 (0.60 – 1.83)	0.29	0.18	0.860
High cholesterol/hyperlipidemia	0.89 (0.76 – 1.06)	0.08	-1.26	0.208
Other circulatory problems	0.82 (0.57 – 1.18)	0.15	-1.04	0.299
Obesity (BMI > 30)	1.10 (0.96 – 1.28)	0.08	1.37	0.172
VA health care use (51 – 100%)	0.91 (0.76 – 1.08)	0.08	-1.03	0.303
Overnight hospital visits (1 or more)	1.06 (0.88 – 1.28)	0.10	0.60	0.546
Rx medications (1 or more)	0.83 (0.66 – 1.03)	0.09	-1.68	0.093
Non-Rx medications (1 or more)	0.91 (0.76 – 1.08)	0.08	-1.10	0.271

Logistic regression was used to estimate the odds of being classified into the CTBIE^{+} group as a function of health outcomes, adjusting for covariates (age, sex/gender, race/ethnicity, and PTSD diagnosis). The Screen^{+}/CTBIE^{-} group served as the reference group. *OR* odds ratio, *CTBIE* Comprehensive Traumatic Brain Injury Evaluation, *TBI* traumatic brain injury, *PTSD* posttraumatic stress disorder, *VR-12* veterans RAND 12 Item Health Survey, *BMI* body mass index, *VA* Veterans Affairs, *Rx* prescription, *CI* confidence interval
